# The development of T cells from stem cells in mice and humans

**DOI:** 10.4155/fsoa-2016-0095

**Published:** 2017-03-16

**Authors:** Farbod Famili, Anna-Sophia Wiekmeijer, Frank JT Staal

**Affiliations:** 1 Department of Immunohematology & Blood Transfusion, Leiden University Medical Center, Leiden, The Netherlands

**Keywords:** HSC, Notch, SCID, thymus, T lymphocyte, Wnt

## Abstract

T cells develop from hematopoietic stem cells in the specialized microenvironment of the thymus. The main transcriptional players of T-cell differentiation such as Notch, Tcf-1, Gata3 and Bcl11b have been identified, but their role and regulation are not yet completely understood. In humans, functional experiments on T-cell development have traditionally been rather difficult to perform, but novel *in vitro* culture systems and *in vivo* xenograft models have allowed detailed studies on human T-cell development. Recent work has allowed the use of human severe combined immunodeficiency stem cells to unravel developmental checkpoints for human thymocyte development.

## Lay abstract

T lymphocytes (often simply termed as T cells) are the main disease-fighting cells in the blood. All blood cells are derived from rare cells in the bone marrow that can self-renew: the blood-forming stem cells. While most types of blood cells develop in the bone marrow from these stem cells, T cells develop in a specialized organ called the thymus. Over the last 20 years, researchers have made tremendous progress in understanding the signals that drive the development of T cells, first in mice, but increasingly also in humans. This is important as in many diseases, T cells and their birth in the thymus play an important role. Better insight into this process therefore directly contributes to better human health.

## Hematopoietic stem cells as a source for all blood cells

The blood in our body consists of many different cell types. Hematopoietic stem cells (HSCs), which reside in the bone marrow (BM), are able to produce all the different cells present in our blood system, including platelets, red blood cells and white blood cells. This involves a highly controlled process of both self-renewal, to maintain the pool of HSCs, and differentiation. The process of both self-renewal and differentiation is coordinated by many signaling pathways, such as Notch [1], Wnt [2], bone morphogenic protein (BMP) [3] and several others [4]. Aberrancies in genes and their expressions, either congenital or acquired, can influence these processes, eventually leading to arrests in development or to the development of hematological malignancies.

Under normal circumstances, HSCs give rise to all white blood cells, including both innate and adaptive immune cells. The innate immune system is already present at birth and is a nonspecific defense against pathogens, and therefore is able to respond quickly. It is comprised of different cells types, including mast cells, macrophages, neutrophils, eosinophils, dendritic cells (DCs) and natural killer (NK) cells. The cells of the adaptive immune system are comprised of B cells and T cells, and are present at birth as the cells from the innate immune system. However, cells from the adaptive immune system respond in an antigen-specific manner. These cells express receptors specific for antigens, and upon antigen encounter they will proliferate but also form memory cells. These memory cells are able to respond quickly upon a second encounter with the same antigen, a regimen that is made use of vaccination, thereby providing protection against the pathogen. The adaptive immune system is only found in vertebrates [5–7].

HSCs are rare cells that are difficult to characterize precisely by marker expression alone. The most robust criterion to determine true stem cell potential is the ability to provide long-term repopulation of an entire host with all hematopoietic lineages [8]. In mice, this is often assessed by performing transplantations in secondary recipients to determine self-renewal capacity [9,10]. For human HSCs, this is, of course, not feasible in a clinical setting. Murine HSCs are characterized by the expression of Sca-1, C-kit, CD38, low expression levels of the Thy-1, low to absent CD34 and the lack of lineage markers (B220, Mac-1, Gr-1, CD3, CD4, CD8 and Ter119). The most widely used HSC population in mouse is the so-called LSK population: lineage marker negative, Sca-1+ and C-kit+. Within this population at least three subsets can be distinguished, namely, long-term [11–14] and short-term [12] HSCs, often by using CD34 in combination with the FLT3 marker and the so-called multipotent progenitors (MPPs) that have largely lost true self-renewal capacity. Other markers are continuously evaluated and added in an attempt to more precisely define true HSCs. Of note are the so-called SLAM markers CD50 and CD48, which further subdivide the LSK population into cells enriched for long-term or short term repopulating stem cells and MPPs [15,16].

## Human HSCs & their clinical use

The regenerative capacity of HSC is of great use in the clinic for the treatment of many diseases affecting the blood system; leukemia, lymphoma, severe combined immunodeficiency (SCID) and hemoglobinopathies, encompassing thalassemia and sickle cell disease [17]. Either autologous or allogeneic stem cells are used for transplantation, often depending on the availability of the donor material. As a first step in the transplantation procedure, the cells of the immune system in the patient are often depleted by chemotherapy, which is called conditioning, and then the patient will receive donor-derived HSC that can engraft and develop a new healthy immune system. HSCs can be isolated from different sources; BM, mobilized peripheral blood and umbilical cord blood, all of which are used in the clinic for transplantation [18].

**Figure 1.**
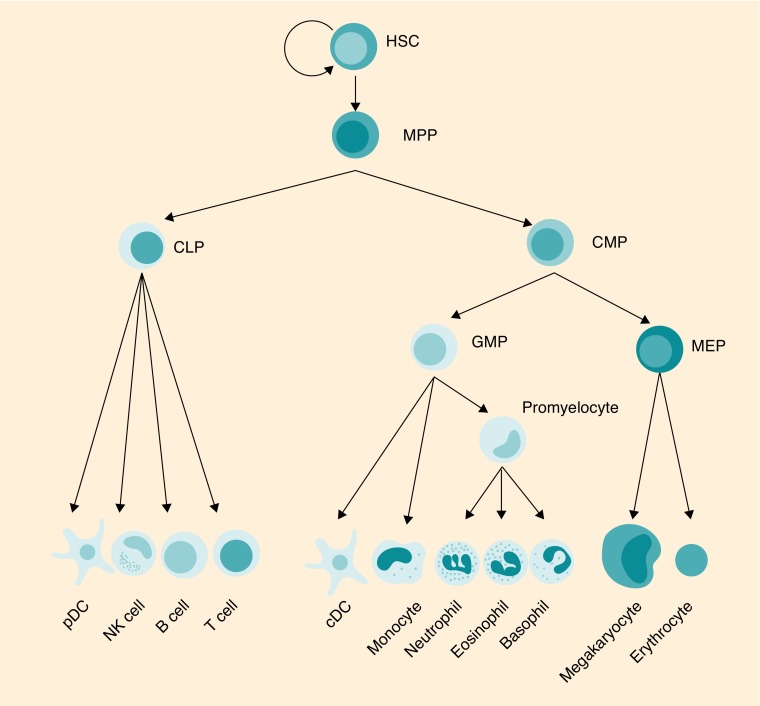
**Schematic representation of the main lineage commitment steps in hematopoiesis.** HSCs with the self-renewal capacity are placed at the top of the hierarchy, develop to several MPPs which give rise to mature blood cells through a step-wise process of lineage commitment. cDC: Conventional dendritic cell; CLP: Common lymphoid progenitor; CMP: Common myeloid progenitor; GMP: Granulocyte-monocyte progenitor; HSC: Hematopoietic stem cell; ILC: Innate lymphoid cell; MEP: Megakaryocytic-erythroid progenitor; MPP: Multipotent progenitor; pDC: Plasmacytoid dendritic cell.

In a clinical setting, the CD34^+^ fraction is used for transplantation as these cells can be isolated in a GLP setting. However, already in 1997 it was described that the phenotype of HSCs could be further refined to CD34^+^CD38^-^ containing a frequency of 1 in 617 cells with true HSC potential, defined by the capacity to repopulate an NOD/Scid mouse [19]. Thereafter, it was shown that this cell fraction can be divided into three groups based on the expression of both CD90 and CD45RA. The Lin^-^CD34^+^CD38^-^CD90^+^CD45RA^-^ cell population isolated from umbilical cord blood was demonstrated to have multilineage BM engraftment potential when ten cells were transplanted [20]. This cell population could be further subdivided by CD49f discrimination of which the CD49f^+^ population contained a frequency of LT (long-term repopulating)-HSC of 1 in 10.5 cells [21]. This illustrates that currently we are not yet able to identify the one cell phenotype that is most primitive and contains the highest long-term repopulating capacity. Currently, the human HSC is described to be most enriched within the Lin^-^CD34^+^CD38^-^CD45RA^-^CD90^+^CD49f^+^ population followed by the MPP that has lost expression of both CD90 and CD49f (Figure 1) [22]. From the MPPs, two cell types branch off: the CD34^+^CD38^-^CD45RA^+^CD90^-^ multilymphoid progenitor (MLP) that can give rise to NK, B and T cells, and the Lin^-^CD34^+^CD38^+^CD45RA^-^CD135^+^ common myeloid progenitor that can give rise to both megakaryocytic-erythroid progenitor and granulocyte-monocyte progenitor [22,23]. The MLP is similar to a common lymphoid progenitor, i.e., a progenitor proposed to be a precursor for lymphocytes but not for myeloid cells [24–26]. In humans, this has been studied less extensively. The MLP comprises mostly lymphoid restricted cells, but also has some myeloid developmental potential; hence, it cannot be considered as a human counterpart of the mouse common myeloid progenitor. Instead, this population seems closer to lymphoid-primed multipotent progenitors, the so-called LMPPs [27].

Importantly, recent insights have identified a novel lineage of lymphocytes, the innate lymphoid cells (ILC) that do not express antigen-specific receptors but share many other properties with T cells [28–30]. Three subgroups are commonly distinguished on the basis of cytokines they produce and the transcription factors required for their development, a characteristic they also share with specific T-cell subsets. The classical NK cells are now referred to as part of the ILC1 cells and lymphoid tissue inducer cells belong to the ILC3 family.

Cells from the myeloid lineage, erythrocytes and granulocytes are progeny from the megakaryocytic-erythroid and granulocyte-monocyte progenitor types. Also on the gene expression level, there is a separation between the lymphoid fate and myeloid fate at the MLP stage [31]. Many of the transcription factors that are important in HSCs are known to be causative of leukemia when deregulated, for example, RUNX1, MLL, SCL/TAL1 and LMO2 [32,33].

Under homeostatic conditions, the number of stem cells has to stay constant, which can be achieved by asymmetric cell division, through which one daughter cell keeps the stem cell identity and the other differentiates. The mechanism regulating asymmetric versus symmetric remains poorly understood in mammals, particularly in HSCs. Depending on their localization, HSCs can divide symmetrically to expand the HSC pool (for instance, during embryonic life in the fetal liver) or asymmetrically [34–36].

Adult HSCs are mostly quiescent. Indeed, most of the true stem cell activity is present in dormant LT-HSCs [37,38]. Additionally, the activation of dormant stem cells, for instance, by inflammatory signals, such as interferons, seems to be reversible as cycling HSCs return to the dormancy upon re-attainment of homeostasis [38]. Dormancy is thought to be a protective mechanism against exhaustion of HSCs, despite self-renewal properties, as there might be a limited self-renewal potential [39].

## T-cell development in the thymus

T-cell development occurs in the thymus, while all other blood cell lineages develop in the BM. The thymus is a bilobed organ located behind the sternum, above the heart. It is significantly large at birth, but the volume of true thymic tissue decreases by ageing, during a process called thymic involution [40,41]. Organogenesis of the murine thymus starts at E10.5. Bilateral endodermal proliferations of the third pharyngeal pouch invade the underlying mesenchyme to form the thymic primordium or anlage [42]. In humans, this starts at the end of the fourth week of gestation. At E12.5 (4–7 weeks in men), the primordia separate from the pharynx and migrate to their definitive location, where they fuse to form a single organ [43,44]. The importance of the thymus as an essential microenvironment for T-cell development is proved by children suffering from the DiGeorge syndrome, in which the thymus often is completely missing. These children have extremely low number of T cells or even a complete absence of T cells [45].

BM hematopoietic progenitors enter circulation and migrate to the thymus where they commit to the T-cell lineage and further mature to functional T lymphocytes. Since thymic progenitors lose their self-renewal potential, a continuous import of progenitors from the BM is required to maintain T lymphopoiesis [46]. However, upon deprivation of thymus from BM-derived progenitors, the thymus can maintain autonomous T-cell development for several months [47], a turnover process which is regulated by BM progenitor colonization. It has been shown that the thymocyte turnover is regulated by natural cell competition between young BM-derived progenitors and old thymus resident progenitors. When the thymus is relieved from outside competition, intrathymic precursors persist and self-renew, resulting in the development of T-cell acute lymphoblastic leukemia (T-ALL) [33].

**Figure 2.**
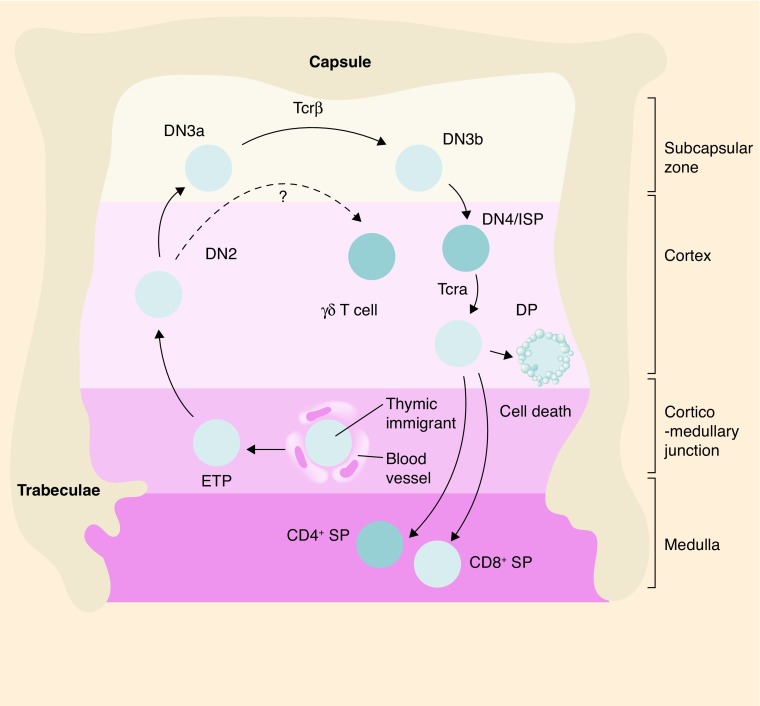
**T-cell developmental stages in the thymus.** Cross section of an adult thymic lobule representing the migration route of T-cell precursors during development. Immigrant precursors move to the thymus through blood vessels and enter near the corticomedullary junction; the ETP subsequently migrate, and differentiate to DN, DP and finally to SP stages, through the discrete microenvironments of the thymus. β-selection occurs at DN3a to DN3b transitions at the outer portion of the thymus (subcapsular zone). A directional reversal of migration back across the cortex toward the medulla occurs for the later stages of thymocyte development. DN: Double negative; DP: Double positive; ETP: Early thymic progenitor; ISP: Immature single positive; SP: Single positive; TCR: T-cell receptor.

T-cell development proceeds through a series of discrete phenotypic stages that can be characterized by the expression of several important membrane molecules, most notably CD4 and CD8 (Figure 2). In both humans and mice, thymocyte development occurs through successive CD4^-^CD8^-^ (double negative, DN), CD4^+^CD8^+^ (double positive, DP) and CD4^+^CD8^-^CD3^+^ or CD8^+^CD4^-^CD3^+^ (single positive, SP) stages. The DN subset can be further subdivided into four stages (DN1 to DN4) in mice and humans [48–50].

The precise identity of the progenitors that seed the thymus is still controversial due to the fact that heterogeneous subpopulations of BM progenitors contain T-cell lineage potential and also the extremely low number of cells seeding the thymus. These progenitors enter the thymus via veins in the cortical tissue close to the corticomedullary junction, from which they migrate into the thymic tissue (Figure 2) [51]. Although a few progenitors migrate to the thymus, they significantly proliferate in response to the environmental signals they encounter, while starting a T-cell transcriptional program. These initial signals are provided via cytokines like stem cell factor (SCF) and Flt3L, Wnt and Notch signaling pathway.

Several types of progenitors have been suggested to seed the thymus, and most of them are known to circulate and express chemokine receptors and adhesion molecules, shown to be involved in thymus migration and seeding. These consist of Ccr7, Ccr9 and the P-selectin ligand Psgl1, among others. Despite several subpopulation candidates of thymus settling, one major progenitor source contains LMPPs [52,53]. These progenitors are defined as Lin^-^ Sca1^+^ c-Kit^+^ Flt3^+^ and, besides T-cell potential, they can develop into macrophages, DCs, NK cells and B cells, but not into erythrocytes or megakaryocyte lineages [54–56], at least in the mouse. In humans, the earliest cells in the thymus have retained some erythroid potential [50], which is also reflected in the abundant erythroid gene program that is expressed in human ETP-ALL [57].

After entering, the thymus T-cell precursors develop through distinct stages. Progression through these steps involves gradual phases of lineage specification, characterized by the acquisition of a T-cell-specific transcriptional program. Concomitantly to the lineage specification events, T-cell precursors gradually and irreversibly lose alternative non-T-lineage potential till they are fully committed to the T-cell lineage. While B-cell potential is rapidly lost by the majority of the progenitors entering the thymus, the potential to become DCs, NK cells and macrophages is preserved until later stages. Initial studies identified cells with a CD3^-^CD4^-^CD8^-^CD25^-^CD44^+^ surface phenotype (named DN1) as the most immature T-cell progenitors in the thymus. Further studies demonstrated that this is still a highly heterogeneous population also containing mature NK, NKT cells and γδ T cells. In addition, the effective T-lineage progenitor activity was shown to reside in a small subset of DN1 cells expressing c-Kit, which was termed early thymic progenitors (ETP) [24,58–60]. ETPs are very efficient in the generation of DN2 cells (defined as CD3^-^CD4^-^CD8^-^CD25^+^CD44^+^) but they still maintain NK, DC, myeloid and, at a lower extent, also B-cell potential. Similar to the LMPPs, a small portion of ETPs also express Flt3 and CCR9. These are believed to be the most immature T-cell progenitors in the thymus in the mouse.

The presence of alternative lineage potential in T-cell progenitors suggests the existence of mechanisms to dictate the T-cell fate at the expense of other lineages. The most well-known instructive signals to promote T-cell development are the Notch signals. Activation of the Notch signaling pathway by ligands of the δ family was shown to be essential to induce T-cell commitment [61–65]. While Notch signaling appears to be involved in the restriction of alternative lineage potentials, for example, by inhibiting B-cell and myeloid cell development, it may alternatively promote survival and expansion of the T-cell progenitor populations [66–68]. Another signaling pathway shown to be essential for these early events in T-cell development is the Wnt signaling pathway, which is currently seen as a rate-limiting positive regulator of the transition to the DN2 stage [69,70].

**Figure 3.**
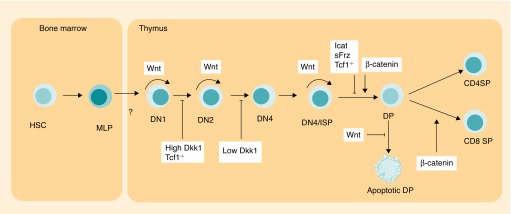
**Effect of Wnt signaling during thymopoiesis in the thymus.** The first cells to arrive in the thymus are rare progenitor cells commonly referred to as ETPs, which reside in the DN (CD4^-^CD8^-^) compartment. DN cells proliferate rapidly, partly mediated by Wnt signaling. Inhibition of the Wnt pathway by ectopic expression of the soluble Frizzled receptor (which acts as a decoy receptor), Dkk1 (which inhibits binding to Ldl receptor-related protein (Lrp co-receptors) or the cell autonomous ICAT (which disrupts the β-catenin–Tcf interaction) leads to inhibition of T-cell development at various points in the DN developmental pathway. Similarly, incomplete blocks in T-cell development are observed at DN1, DN2 and ISP stages of development in Tcf1-deficient mice. Wnt signaling also regulates the survival of DP (CD4^+^CD8^+^) thymocytes by upregulating expression of the antiapoptotic protein Bcl-Xl, and stabilized β-catenin affects positive selection and IL-7 receptor signaling, results in the increased number of CD8^+^ SP thymocytes. Moreover, the levels of CD4 on both DP and CD4^+^ SP cells are regulated in part by Tcf1 (not shown in the figure). Dkk1: Dickkopf homolog 1; DN: Double negative; DP: Double positive; HSC: Hematopoietic stem cell; ICAT: Inhibitor of β-catenin and Tcf; ISP: Immature single positive; MLP: Multilineage progenitor; SP: Single positive; Tcf1: T-cell factor 1. Adapted with permission from [76].

T-cell progenitors migrate through different anatomical zones in the thymus which may provide different signals to help the establishment of a T-cell development program (Figure 3) [71,72]. As ETPs are migrating through the cortical region toward the subcapsular zone, they become more restricted to the T-cell lineage and start expressing important genes for T-cell receptor (TCR) rearrangements, assembly and signaling, such as recombinase activating gene 1 (Rag1) and Rag2, CD3 chains and Lck [48]. The progressive upregulation of T-cell identity genes is accompanied by the acquisition of DN2 (CD3^-^CD4^-^CD8^-^CD25^+^CD44^+^) and DN3 (CD3^-^CD4^-^CD8^-^CD25^+^CD44^-^) surface phenotypes, a process called T-cell commitment. The T-cell commitment process has been studied more extensively, which resulted in the identification of more intermediate stages namely DN2a, DN2b, DN3a and DN3b. DN2a T cells also express high level of c-kit and are believed to be the last uncommitted stage of T-cell development. The expressions of c-kit diminish significantly at the DN2b stage while they lose their capacity to differentiate into any non-T-cell lineage anymore [73]. CD27 expression subdivides DN3a and DN3b pre- and postselection DN3 cells, respectively. Detailed gene expression analysis revealed that regulatory changes associated with the β-selection occur between these two stages of DN3 [74]. The DN3a stage is characterized by an arrest in the cell cycle allowing, in this way, the rearrangement of the *Tcrb* genes that encode for the variable region of the antigen receptors in T cells. These rearrangements occur through a process termed V(D)J recombination which allows the generation of a high diversity of antigen receptors [75]. Successful rearrangement of the *Tcrb* gene is functionally tested by its expression on the cell membrane. Productively rearranged Tcrβ chains are coupled to an invariant pre-Tα chain to form the Pre-TCR complex.

Signaling through the Pre-TCR induces proliferation, survival and differentiation, in a process called β-selection. Cells that pass β-selection are educated to develop into the αβ-T-cell lineage [77] and subsequently develop into DN4 (CD3^-^CD4^-^CD8^-^CD25^+^CD44^-^), ISP (CD3^-^CD4^-^CD8^+^ in mice or CD3^-^CD4^+^CD8^-^ in humans) and DP (CD4^+^CD8^+^) surface phenotypes. After these highly proliferative stages, another arrest in proliferation happens when the cells reach the DP stage and start rearranging the *Tcra* gene. Efficient *Tcra* rearrangement leads to the expression of a TCRαβ complex on the cell surface. These TCRαβ complexes are then functionally tested for the recognition of self-MHC molecules (positive selection) and the absence of reactivity against self-antigens (negative selection) [78].

Therefore, this stage is identified by high apoptosis rate in order to eliminate nonfunctional and autoreactive T cells [79]. Concurrently with the positive and negative selection processes, cells with a functional TCR further maturate to CD4^+^ T-helper cell or to CD8^+^ cytotoxic T-cell lineages and migrate to the periphery.

## Notch & Wnt signaling pathway in the thymus

### Notch signaling

Hematopoiesis and thymopoiesis, like other developmental processes, require a strict spatial and temporal controls, and harmonized gene expression programs. The majority of lineage commitment events in metazoans are controlled by merely a few signaling pathways including Wnt, Notch, TGF-β, Hedgehog and receptor tyrosine kinases. Each pathway is frequently used in several processes, activating diverse subsets of target genes in various developmental contexts.

The Notch signal transduction pathway is not unique to developing T cells, but in the development of blood cells its most prominent role is to induce a T-cell gene program in MPP cells that arrive in the thymus [1]. In many other tissues and organs, Notch signaling similarly regulates cell fate determination. Notch signaling involves cell–cell interactions, rather than binding of a soluble ligand to a receptor. There are four Notch receptors, named Notch1–4. Signaling is initiated when the large extracellular domain of the Notch receptor binds a membrane bound ligand on a neighboring cell. The five Notch ligands in mammals are Delta-like 1, 3 and 4, and Jagged1 and 2. Delta-like 2 is a nonexpressed pseudogene. Interaction of Notch with a ligand induces proteolytic cleavage of the intracellular (IC) part of Notch (IC-Notch). IC-Notch then translocates to the nucleus and binds to the nuclear transcription factor CSL (CBF1 stands for core-promoter binding factor in humans, suppressor of hairless in *Drosphila*, Lag-1 in *Caenorhabditis elegans;* also called RBP-k in mouse). Binding of IC-Notch to CSL induces the dislocation of co-repressors (coR) such as Mint and Nrarp, and recruitment of co-activators (coA), such as Mastermind (Maml), consequently leading to activation of Notch target genes [80].

In the thymus, there is abundant expression of DLL4, the Notch ligand that is mostly responsible for inducing a T-cell lineage program on developing thymocytes [81]. Indeed, Notch1-deficient mice show an arrest in T-cell development at the DN1 stage with a relative increase of B cells in the thymus [65]. Conversely, ectopic expression of IC-Notch in the BM leads to T-cell development in the BM niche with DP cells containing *Tcrb* rearrangements [64].

### Canonical Wnt signaling

The Wnt pathway is yet another evolutionary conserved pathway that is essential for T-cell development after inducing the T-cell program. As the Wnt pathway and its role in T-cell development have been extensively reviewed in a number of review articles [2,76,82], we will here merely make a few remarks on recent insights. For a short overview, Figure 3 serves also as quick reference for the many roles that Wnt signaling plays in thymocyte development.

As the number of progenitor seeding the thymus is limited, an enormous expansion of cells takes place during the early phase of T-cell development. Cytokines, mainly IL-7, but also Wnt proteins, are responsible for the initial proliferation of thymocytes before β-selection. Indeed, it was previously shown that Wnt proteins are secreted by thymic stromal cells, and canonical Wnt signaling is most active in the most immature DN stages [83]. In this regard, it is relevant that the first T-cell-specific target gene of the Notch pathway to be induced is Tcf-1 (encoded by the gene confusingly named *Tcf7*), the nuclear protein responsible for transmitting the nuclear response to Wnt signals. In this way, the Notch pathway starts a positive feedback loop in which T-cell-specific signals are amplified by Wnt signaling via Tcf-1. We have shown before that especially early T cells are ‘hard wired’ to respond to Wnt signals. As this differential responsiveness to Wnt signaling during different thymocyte stages was not caused by altered expression of Frizzled or Wnt proteins, but by increased expression of positively acting canonical Wnt factors (such as β-catenin) and decreased expression of inhibitory molecules (such as Axin1) in early DN thymocytes [83].

As an approach to understand the controversies in the literature on Wnt signaling in HSCs, we previously reported a combination of targeted mutations in *Apc*, which were used in order to obtain a gradient of Wnt signaling activation. While HSC function was enhanced specifically with mild levels of Wnt signaling activity, only intermediate Wnt activation confers an advantage to the early stages of T-cell development. High and very high levels of Wnt signaling activation, similar to stabilization of β-Catenin, resulted in accumulation of DN3 thymocytes and in impaired *Tcrb* gene rearrangements [84]. Despite the severe reduction in *Tcrb* gene rearrangements, Tcrβ-DP and SP cells could be detected in the thymus of these mice, although in reduced numbers, indicating that high Wnt signaling allows a bypass of the β-selection checkpoint. The reduced numbers of DP and SP thymocytes are probably due to the lack of proliferation and survival stimuli from a functional pre-TCR [85,86]. In contrast, an intermediate activation of the Wnt pathway enhanced early stages of T-cell development while preserving *Tcrb* rearrangements and maintaining developmental checkpoints. However, the number of DP cells was still reduced which may indicate that later stages of T-cell development have different Wnt signaling requirements [87]. In agreement, in *in vitro* co-cultures, with OP9 stromal cells expressing the Notch ligand Delta1, in which positive and negative selection processes are less stringent [88], an increase in both DP and SP cells with intermediate but not higher levels of Wnt activation was observed. In summary, HSCs and thymocytes require different levels of Wnt activation with the thymocytes displaying higher Wnt activity. The higher levels of Wnt signaling in HSCs lead to loss of stemness and enhanced differentiation [89].

### Noncanonical Wnt pathway

In addition to the above-described canonical Wnt pathway, the so-called noncanonical pathway that does not involve β-catenin but Wnt-Fz binding induced changes in cytoskeleton and cell migration, sometimes via increases in intracellular-free calcium. The role of the noncanonical Wnt-Ca^2+^ pathway in T-cell development was investigated using mice deficient of Wnt5a, or by providing high levels of exogenous Wnt5a [90]. Thus, noncanonical Wnt signaling in the thymus seems to play an important role in regulating apoptosis [91–93]. Indeed, mice deficient in the noncanonical Wnt receptor Ryk show increased apoptosis, although overall T-cell development is largely unaffected, with the exception of a transient increase in thymic NK cells [94].

## Transcriptional drivers of T-cell commitment

T-cell commitment depends on a collection of various transcription factors, each with its own expression profile including Myb, Runx1 with its partner CBFb, GATA-3, TCF-1 (encoded by *Tcf7*), Bcl11b and E2A (which encodes two alternative splice variants E12 and E47). Weber *et al*. have shown that Tcf-1 is highly expressed in the earliest thymic progenitors, and its expression is upregulated by Notch signals [95]. However, when Tcf-1 is ectopically expressed in BM progenitors, it induces the development of T-lineage cells in the absence of Notch1 signals. Further characterization of these Tcf-1-induced cells showed expression of several T-lineage genes, including T-cell-specific transcription factors Gata3 and Bcl11b, and components of the TCR [95].

In a related study by Germar *et al*., it has been proven that Tcf-1 is required at the earliest phase of T-cell development for progression beyond the early thymic progenitor stage. The earliest deficiency detected in Tcf-1 KO thymocytes was the reduced expression of c-kit at the DN1 stage of development. Tcf-1 KO cells at this stage showed increased apoptosis and have significantly reduced expression of genes involved in DNA metabolic processes, chromatin modification and response to damage compared with their wild-type counterparts [96].

Induction of the T-cell developmental program initially depends on Notch signaling. Notch signaling is required to set up the first T-cell specific genes, initially *Tcf7*, later *Gata3* and also *Bcl11b*, the transcription factor that seals off the T-cell commitment fate [95,97]. Developing T cells initially express a considerable level of PU.1, but inhibit it completely during lineage commitment. This process takes surprisingly long, almost 14 days (at least 10–12 cell divisions) in the mouse and probably longer in humans based on OP9 cultures with human CD34^+^ cells [98]. Thus, these cells have only a short opportunity in which myeloid potential poses a threat to T-lineage fidelity. In fact, the thymic microenvironment is nonpermissive for expression of this myeloid potential [99], although in both mouse and human systems, B and myeloid potential can be detected.

The T-cell program is orchestrated by inducing expression of three transcription factors: Gata3, Tcf7 and the Bcl11b. These transcription factors rely all in part on Notch signaling via RBP-j. Gata-3 is essential for T-cell development from the earliest stage throughout multiple later developmental checkpoints, and it is restricted in its hematopoietic expression to T cells and ILCs [100]. Gata-3 can antagonize the alternative lineage fate through its ability to repress PU.1 and Pax5 [101]. Gata3 expression after initial Notch-dependent induction is probably maintained by Myb and Tcf-1 as likely positive regulators [102].

T-cell commitment completes when thymocytes progress to DN2 stage and then increase expression of the Bcl11b gene [103,104]. Indeed, Bcl11b is turned on by Notch signaling and probably by other factors, and inhibits residual NK cell lineage potential in DN2 cells [99,103].

Recently, Rothenberg and co-workers unraveled the mechanisms of *Bcl11b* activation during T-lineage commitment by generating a knock-in fluorescent reporter at the *Bcl11b* locus and followed *Bcl11b* activation dynamics at the single-cell level using *in vitro* developmental assays together with flow cytometry and time-lapse live imaging [105]. They showed that the factors that are controlling *Bcl11b* expression amplitude differ from those that license the locus for expression competence, a regulatory strategy that enables the latter to have subsequent roles in mature T-cell functional specialization. These factors work via three distinct, asynchronous mechanisms: an early locus ‘poising’ function dependent on TCF-1 and GATA-3, a stochastic-permissively function dependent on Notch signaling and a separate amplitude-control function dependent on Runx1, which is already expressed at the HSC level [105].

Another transcription factor that is involved in inducing rearrangements in both T and B cells is E2A [106,107]. Loss of E2A activity results in a partial block at the earliest stage of T-lineage development [108]. This early T-cell phenotype precedes the development T-cell leukemias [109], as also occurs in thymocytes lacking Tcf-1 [110]. Thus, both E2a and Tcf-1 are not only crucial as positively acting transcription factors, but also as tumor suppressor genes for the development of thymic lymphomas/leukemias.

## Human T-cell development & SCID

In humans, three phenotypes of thymus seeing progenitors (TSP) have been proposed; a CD34^hi^CD45RA^hi^CD7^+^ phenotype [111], Lin^-^CD34^+^CD10^+^CD24^-^ [112] and cells characterized as Lin^-^CD34^+^CD10^-^CD45RA^+^CD62L^hi^ [113]. Haddad *et al*. did show that the CD34^hi^CD45RA^hi^CD7^+^ cells they identified were able to migrate into thymic lobes in an *ex vivo* culture system [111]. These cells could be differentiated from CD34^+^ cells isolated from cord blood using the *in vitro* OP9-DL1 co-culture system and were able to engraft thymi of immune-deficient mice [71]. The TSP phenotypes identified by Six *et al*., however, do not express CD7 and have the capacity to develop into B cells, NK cells and T cells using *in vitro* co-culture systems [112]. Furthermore, human thymus cells positive for the expression of CD34 but negative for the expression of CD7 can be found, and it was demonstrated that CD34^+^ cells upregulate CD7 only after 4 days of co-culture on OP9-DL1 [114]. This argues against CD7^+^ cells as being the most immature cells present in the thymus; however, it might be that the thymus can also be seeded by multiple populations. The cells identified by Kohn *et al*. are negative for the expression of CD7 but they did not succeed in transplanting these cells in immune-deficient mice to monitor thymus seeding and engraftment [113]. Therefore, the nature of the TSP in humans remains controversial.

There are many similarities between T-cell development in mice and men, as in both species it starts in the CD4^-^CD8^-^ DN compartment, and also the CD4^+^CD8^+^ DP stage is comparable after which the cells become either CD4^+^ SP or CD8^+^ SP (Figure 3). The SP cells have undergone positive selection, to select for thymocytes of which their TCR can bind MHC (in humans also called HLA), and negative selection, to eliminate cells that recognize self-antigens. These mature T cells are now ready to emigrate to the peripheral blood.

However, differences also exist between T-cell development in mice and men. Between the DN and DP stages lies the immature single positive (ISP) stage that is CD8^+^ in mice and CD4^+^ in humans, but in both species there is no expression of CD3 or a TCR [115]. In mice, the DN compartment can be further subdivided into DN1–4 based on the expression of CD44 and CD25 [45], and further subdivision of these DN compartments has been described too in [59]. The DN compartment of human-developing T-cell progenitors can also be subdivided, but different markers have been described to do so, for example, CD34 in combination with CD38 and CD1a [48], or CD1a and CD5 and CD7 [114]. During T-cell development, the TCR loci are rearranged; most cells will eventually become a TCRαβ^+^ T cell; and in mice, the point of β-selection is at the DN3 stage. At this point, the progenitors need to have rearranged the *Trb* locus in a way that produces a functional β-chain; otherwise, the progenitor cannot progress in its development and will eventually die [116]. In humans, it has been debated where the point of β-selection exactly resides; it has been ascribed to the DP stage [117], the immature single positive stage [74,116] and to the CD1a^+^ DN3 stage [50]. Until recently, the prevalent opinion was that, as in mice, the β-selection/pre-TCR checkpoint resides in the DN3 population [48,115]. Two recent reports indicate that this developmental checkpoint may occur even earlier in the development. Using CD34^+^ umbilical cord blood progenitor cells seeded on OP9-DL1 cells to induce T-cell development via Notch signaling, Meijerink and co-workers showed that the CD34^+^CD1a^-^ subset can be subdivided into CD44^+^ and CD44^-^ cells, with the CD44^+^ cells having full TCRB rearrangements, higher expression of T-cell linage genes and solely T-cell developmental potential [118]. This identifies CD44 as a marker within the human DN subset for T-lineage commitment. The second study, described in more detail below, used a genetic approach to show that cells lacking Artemis, an essential gene in the recombination complex for TCR and Ig genes (see below), are blocked at the CD7^+^CD5^+^CD1a^-^ stage. Thus, these two studies collectively show that the CD34^+^CD1a^-^ subset, often referred to as human DN3, is heterogeneous. This is analogous to mouse DN3 cells which contain pre- and post-β-selection thymocytes and can be subdivided on the basis of size or CD27 expression.

SCID is a subset of primary immunodeficiency that is characterized by a deficiency in (functional) T cells with an incidence ranging from 1 to 3 per 100,000 live births [119–121]. Patients often present within their first year of life with a failure to thrive and recurrent infections [122]. Different forms of SCID exist in which the deficiency in T cells can be accompanied by a deficiency in B cells or NK cells, or both. As SCID patients lack an adaptive immune response, they present with opportunistic infections and a failure to thrive, which is often diagnosed within their first year of life. The different phenotypes of SCID are caused by the differences in mutations and affected genes that are causative of SCID (Table 1). Currently, around 16 genes have been identified [123,124], but there are still cases of affected children without a known genetic cause. In a cohort studied by Gaspar *et al*. [125,126], there were 20 out of 117 patients (17%) without a molecular diagnosis; this percentage might be different in other cohorts as the presence of certain types of SCID can vary between geographic regions. The different types of SCID are categorized into two different groups based on the presence or absence of B cells: T^-^B^-^ SCID and T^-^B^+^ SCID. Both groups encompass patients with the presence or absence of circulating NK cells, depending on the genetic aberrancy. Recently, we made use of the Nod Scid common gamma (NSG) xenograft model [127] and lentiviral cellular barcoding to study hematopoiesis and T-cell development at a clonal level [128], as well as of CD34^+^ cells from normal human BM or cord blood and of stem cells from different types of human SCID [129].

**Table 1.** **Overview of Severe combined immunodeficiency-causing genes.**

**Phenotype**	**Affected gene**
T^-^B^-^	ADA, AK2 RAG1, RAG2, Artemis (DCLRE1C), DNA-PKcs (PRKDC), LIG4, XLF
T^-^B^+^	IL2RG, JAK3 IL7RA, CD45 (PTPRC), CD3D, CD3E, CD3Z (CD247), CORO1A

SCID is characterized by a deficiency of (functional) T cells that can be accompanied by a deficiency in B cells or NK cells or both. Indicated are the different phenotypes of SCID and the genes that, when mutated, can cause this type of deficiency. Included genes were based on criteria described by Bousfiha *et al*. [123].

NK: Natural killer; SCID: Severe combined immunodeficiency.

We used this model to characterize the blocks in T-cell development for different types of SCID (ADA, Artemis, IL7RA and IL2RG). We observed blocks in T-cell development for IL7RA- and IL2RG-SCID at the CD4^-^CD8^-^ DN stage. A later block but still before the DN CD1a^+^ stage was observed with Artemis-deficient SCID. The Artemis nuclease (gene name *DCLRE1C* [DNA cross-link repair 1C]), in a complex with the DNA-dependent protein kinase (DNA-PKcs), is required to resolve the hairpins that are formed at the coding ends of rearranged gene segments during V(D)J recombination [130,131]. Artemis binds to these DNA ends and makes a single cut near the tip of the hairpin, and is required in both T and B cells’ development in the recombinase machinery. In a study by Wiekmeijer *et al*., *TCRB* rearrangements were initiated much earlier than previously thought, as determined from the developmental block and the extent of rearrangements observed in mice transplanted with Artemis-SCID HSC [129]. These data provide previously unattainable insight into human T-cell development using SCID as human loss-of-function models. Moreover, they indicate an even earlier β-selection point in human T-cell development.

We have also used this model to address a fundamental problem in T-cell development, namely, how many stem cell clones contribute to the T-cell linage. Given the spatially different developmental locations for T cells versus all other blood cell lineages, a certain clonal restriction can be expected. We used lentiviral cellular bar coding with purified human HSCs transplanted into NSG mice to address this question [128]. Barcoded HSCs showed reproducible myeloid and lymphoid engraftment. Of the many transplanted HSC clones, only a limited number (<10) repopulated the thymus, with a further restriction of the number of clones at the DN stages. Despite this restriction in the stem cells contributing to the T-cell lineage, TCR rearrangements were polyclonal and a diverse, showing that a multitude of T-lymphocyte clones with different specificities can develop from a single HSC clone [128]. Thus, even a single HSC clone can give rise to a fully diverse TCR repertoire.

## Conclusion & future perspective

The mouse has been the experimental model system of choice for immunologists and hematologists working on HSCs and development of T lymphocytes. However, in recent years much attention has also been devoted to the study of human immune system [132,133]. With novel molecular tools such as RNA-Seq and possibilities of using advanced genetic techniques such as the CRIPSR-CAS9 system (allowing loss-of-function studies on human cells) coupled with *in vivo* and *in vitro* xenotransplantation models (e.g., OP9-DL1 [134,135], NSG [127,136], NOG), the study of human hematopoiesis and T-cell development can now also be approached via mechanistic experiments. Over the next few years, we will likely see a full understanding of the transcriptional hierarchy driving T-cell development in the mouse, and this body of knowledge will increasingly be directly tested in human stem cells and thymocyte differentiation schemes. This is important; despite many fundamental similarities, significant differences between mice and humans exist in the stem cell compartment and thymus alike, necessitating the study of the human immune system not only to understand our own species but also to translate findings into clinical treatment modalities. As the thymus is not only involved in rare diseases such as SCID and DiGeorge syndrome but also is the target of more common viral infections (e.g., HIV disease), directly involved in autoimmune diseases, and involuted during ageing, leading to senescent T-cell responses, better knowledge of human T-cell development (often using concepts first discovered in the mouse system) is crucial in a wide variety of diseases and conditions.

**Executive summary**

- Multilineage differentiation and self-renewal ofhematopoietic stem cells (HSCs are controlled by several conserved signaling pathways.
- Human and mouse HSCs have been defined by different phenotypic markers allowing their prospective isolation.
- Human HSCs are clinically used to treat hematological malignancies and as target cells for gene therapy.
- Notch signals are crucial to induce T-cell development; Wnt signals provide essential proliferative signals to the most immature thymocytes.
- In the mouse, major transcription factors that underlie T-cell development include E2A, Tcf1, Gata3 and Bcl11b.
- Human severe combined immunodeficiency can be used as the loss-of-function model for studying human T-cell development.
